# Cost-effectiveness of providing measles vaccination to all children in Guinea-Bissau

**DOI:** 10.1080/16549716.2017.1329968

**Published:** 2017-06-05

**Authors:** Stine Byberg, Ane Bærent Fisker, Sanne Marie Thysen, Amabelia Rodrigues, Ulrika Enemark, Peter Aaby, Christine Stabell Benn, Ulla Kou Griffiths

**Affiliations:** ^a^ Bandim Health Project, Indepth Network, Bissau Codex, Guinea-Bissau; ^b^ Research Center for Vitamins and Vaccines (CVIVA), Statens Serum Institut, Copenhagen S, Denmark; ^c^ OPEN, Odense Patient data Explorative Network, Odense University Hospital/Institute of Clinical Research, University of Southern Denmark, Odense, Denmark; ^d^ Department of Public Health, Centre for Global Health, Aarhus University, Aarhus, Denmark; ^e^ Department of Global Health and Development, London School of Hygiene and Tropical Medicine, London, UK

**Keywords:** Measles vaccine, cost-effectiveness, non-specific effects, barriers to vaccination

## Abstract

**Background**: Measles vaccination is associated with major reductions in child mortality and morbidity. In Guinea-Bissau, to limit vaccine wastage, children are only measles-vaccinated if at least six children aged 9–11 months are present at a vaccination session.

**Objective**: To estimate the incremental cost-effectiveness of providing measles vaccine (MV) to all children regardless of age and number of children present.

**Methods**: We estimated MV coverage among children living in villages cluster-randomized to MV for all children and among children cluster-randomized to the current restrictive MV policy (status quo). Prices of MV and injection equipment were obtained from the United Nations Children’s Fund (UNICEF). Cost savings of hospital admissions averted were collected from a sample of health facilities. The non-specific mortality effects of MV were estimated and presented as deaths averted and life years gained (LYG) from providing MV-for-all.

**Results**: MV coverage at 36 months was 97% in MV-for-all clusters and 84% in restrictive MV policy clusters. Conservatively assuming 90% wastage of MV under the MV-for-all policy and 40% under the restrictive MV policy, cost per child vaccinated was USD 3.08 and USD 1.19, respectively. The incremental costs per LYG and death averted of the MV-for-all policy were USD 5.61 and USD 148, respectively. The MV-for-all policy became cost-saving at 88% wastage.

**Conclusions**: Taking the low cost of MV and the beneficial non-specific effects of MV into consideration, a 10-dose MV vial should be reclassified as a ‘1+ dose vial’. The vial should be opened for a single child, irrespective of age, but can vaccinate up to 10 children.

## Background

Since the introduction of measles vaccine (MV) in the routine vaccination programme in Guinea-Bissau in the early 1980s, measles-related mortality and morbidity have decreased markedly [1]. The effectiveness of MV in preventing measles infection and measles sequelae has been well documented; in children older than nine months of age, at least 85% of those vaccinated are protected against measles after the first dose [2,3]. Measles is however still the leading cause of vaccine-preventable deaths in children and the fifth leading cause of death in children under five years of age, which makes adequate measles control imperative in the effort to lower child mortality [4].

The cost-effectiveness of MV has been assessed in several studies; in a systematic review of cost-effectiveness of vaccines in low- and middle-income countries, MV was associated with cost-effectiveness ratios of between USD 1.5 and USD 240 per Disability Adjusted Life Year (DALY) averted [5]. In a study from Benin, the cost-effectiveness of routine MV was USD 1.60 per DALY averted [6]. The average cost-effectiveness of increasing coverage of MV to 80–95% across countries in sub-Saharan Africa (World Health Organization [WHO] Afr-E region) was estimated to be approximately 25 international USD per DALY averted [7]. Although MV is already deemed highly cost-effective in these studies, benefits may be underestimated. MV reduces all-cause morbidity and mortality much more than can be explained by measles protection alone [8–11]. In a recent WHO-commissioned review of these so-called ‘non-specific effects’ of MV in low-income countries, the vaccine was associated with reductions in all-cause child mortality of up to 46% (95% confidence interval [CI]: 35–55%) [12].

In Guinea-Bissau in West Africa, routine vaccinations are provided free of charge at health centres and through outreach services. Nonetheless, MV coverage rates of the first dose of MV in Guinea-Bissau are sub-optimal with an estimated coverage of 69% in 2014 [13]. Thus, one-third of children older than 12 months have not received MV. The WHO/UNICEF (United Nations Children’s Fund) Joint Reporting Form instructs the Ministry of Health to report vaccination coverage among infants only. Hence, vaccination of children older than 12 months does not count in the assessment of coverage, which is calculated as the number of doses administered to children < 12 months divided by the estimated target population. Consequently, although the WHO recommends that MV should not be limited to children aged 9–11 months [2], vaccinating children older than 12 months of age in Guinea-Bissau is considered wastage. Furthermore, the lyophilized, live MV comes in 10-dose vials, which has to be used within 6 hours after dilution. Increased focus on wastage of vaccine doses has made it more difficult to obtain vaccination. While the MV target wastage rate was 15% in 2010, it was 11% in 2014 [14]. Thus, to meet the target wastage rate of 11%, a local policy has been implemented where MV vials are only opened if six or more children 9–11 months of age are present at the same time (‘restrictive MV policy’). These very low wastage targets have also been specified in other African countries [15], indicating that restrictions on vial opening may exist in several other countries. In rural Guinea-Bissau, this restrictive MV policy has resulted in several missed measles vaccination opportunities and an increased cost burden to households [16].

Taking the non-specific effects of MV into account, we aimed to assess the incremental cost-effectiveness of an MV-for-all policy where children are measles-vaccinated regardless of age and number of children present, compared with the current restrictive MV policy (status quo).

## Methods

### Setting

The Bandim Health Project (BHP) has conducted research based on a routine demographic surveillance system in Guinea-Bissau since 1978. The BHP follows 182 clusters of approximately 100 women of fertile age and their children under five years of age in the nine rural health regions of Guinea-Bissau. The clusters were initially selected using the methodology used by the Expanded Programme on Immunization (EPI) for immunization surveys. Every six months, the clusters are visited by BHP mobile teams for registration of new pregnancies and children. The BHP collects information aimed at assessing the health of all children below five years of age. At every visit, information on vaccinations is obtained by inspecting the child’s health card and the date of the vaccination is noted. A BHP nurse accompanies the mobile teams and offers routine vaccines to children who are missing vaccinations. At all visits, the caretaker is interviewed about hospital admissions. If the child has been admitted to hospital since the last visit, a short questionnaire with information on hospital admission date, location and duration is completed.

We used costs and effect estimates derived from studies and registers from the BHP as well as public sources, described in further detail in what follows. Costs were estimated from a societal perspective.

### The MVEPI study

In February 2011, BHP initiated a cluster-randomized trial aimed at evaluating the current ‘restrictive MV policy’, the MVEPI study. The trial studied the effect on mortality, morbidity and growth of providing MV for all children regardless of age and number of children present. The reporting of the results of the trial is forthcoming. All 182 rural clusters under survey by BHP were assigned a number, and using STATA to generate random numbers, all clusters were randomized to either ‘MV-for-all’ or ‘restrictive MV policy’. During the biannual visits by the BHP mobile teams, measles-unvaccinated children between 9–36 months of age were eligible for enrolment. Measles-unvaccinated children in the restrictive MV policy clusters were vaccinated by the BHP if they were aged 9–11 months at the visit and if six or more measles-unvaccinated children aged 9–11 months were present at the same time. In the MV-for-all clusters, all measles-unvaccinated children aged 9–36 months were measles-vaccinated by the BHP regardless of number of children present. Throughout the MVEPI study there were no reported cases of measles in Guinea-Bissau [17].

### Study population

We estimated costs and effects for the 2011 national birth cohort in Guinea-Bissau, comprising 59,062 children [14]. Using the distribution of deaths from the 2011 rural birth cohort under BHP routine surveillance, we calculated the percentage of children who died between 0–9 months of age and estimated the number of children alive to receive MV at 9 months in the national birth cohort (Table 1).

**Table 1.** Model parameters.

Variable used	Value	Source of information [reference]
2011 birth cohort (number of children)	59,062	[14]
Percentage of the 2011 birth cohort under surveillance by BHP who died before 9 months of age	7.6%	BHP surveillance data
2011 birth cohort surviving until 9 months of age (number of children)	54,573	
Hospital admission rate among children aged 9–48 months per 1000 person years (restrictive MV clusters)	12.5	BHP routine surveillance data
Hospital admission rate among children aged 9–48 months per 1000 person years (MV-for-all clusters)	8.9	BHP routine surveillance data
Mean duration of hospital admission (days)	7.6	BHP routine surveillance data
Cost per hospital bed-day (USD)	14.97	[18]
MV-associated mortality reduction	46%	[12]
Household costs of accompanying a child to hospital per day (USD)	2.8	[19]
Life expectancy in Guinea-Bissau (years)	55	[20]
Infant mortality rate (not including neonates) in Guinea-Bissau	47.6/1000	[21]
Mortality rate 1–4 years in Guinea-Bissau	71.2/1000 (17.8 per year)	[21]

We derived MV coverage from the 2011 BHP birth cohort. MV coverage rates at 12, 24 and 36 months were determined from children with a seen health card between 12–23 months, 24–35 months and 36–47 months of age, respectively. We used the coverage in the restrictive MV policy clusters as a proxy for coverage under the current restrictive MV policy and MV coverage in the MV-for-all clusters as a proxy for coverage under an MV-for-all policy in the 2011 national birth cohort.

### Costs incurred by the health system

We assumed that the only MV-related costs that would differ between the two policies were the costs of extra vaccine vials, syringes and safety boxes. Thus, we assumed that health centre staff would not spend extra time on vaccination in the MV-for-all clusters compared with the restrictive MV policy clusters, as staff also spend time telling mothers to come back another day under the restrictive MV policy. The cold chain equipment has recently been upgraded in Guinea-Bissau so that all primary health care centres have solar fridges with space for extra vaccines. We therefore assumed that it was not necessary to acquire extra cold chain equipment to provide MV for all.

For calculating the number of vials used under the current restrictive MV policy, we assumed that 6 children were vaccinated per 10-dose vial, equalling 40% wastage. Under the MV-for-all policy, as a worst-case scenario, we assumed one vial per child vaccinated (90% wastage). We also calculated the median number of children vaccinated per MV vial in the MV-for-all clusters.

We used the 2015 UNICEF price for a 10-dose measles vial of 2.65 USD [22]. The price of one 0.5 ml syringe used for administering MV was USD 0.05 and a 5 litre safety box was USD 0.54 [23]. The price of a 5 ml syringe used for diluting the vaccine was USD 0.04 [24]. We assumed one 0.5 ml syringe per child vaccinated, one safety box per 100 children vaccinated and one 5 ml syringe per MV vial used. We assumed 5% wastage of syringes.

### Hospital admissions

We estimated the hospital admission rates per 1000 person years for children under surveillance by the BHP, aged 9–48 months born in 2011, based on maternal reports of hospital admissions. The estimates were calculated separately for children residing in the restrictive MV policy clusters and in the MV-for-all clusters.

We applied the hospital admission rates to the number of person years in the 2011 national birth cohort. We assessed the mean number of bed days among children 9–48 months of age who were admitted to hospital and under survey by the BHP. The costs of a hospital bed day were estimated to be USD 14.97 in 2013 [18]. We calculated the costs saved from averted hospital admissions due to higher MV coverage in the MV-for-all scenario, by multiplying the difference in hospital admissions between the two policies by the mean admission duration and by the costs of a hospital bed day. The exact duration of the non-specific effects of MV is unknown. Thus, future costs of hospital admissions averted beyond 48 months of age were not taken into account.

### Costs incurred by families

The value of the mother’s time spent on accompanying her child to hospital was determined according to the average earnings across all sectors in the economy. Based on data on yearly earnings from 36 countries, Knight et al. constructed a regression model with Gross National Income (GNI) per capita as the independent variable [19]. For Guinea-Bissau this model estimated average monthly earnings as 61 USD in 2011 [19]. We converted this to USD 2.8 per day, assuming 22 working days per month. We multiplied the value of the mothers’ time by the mean duration of bed days per hospital admission and by the number of admissions averted due to higher MV coverage.

In a previous study from rural Guinea-Bissau, mothers on average took their children for MV 1.4 times before succeeding in getting the child vaccinated under the current restrictive MV policy [16]. Thus, we added these costs incurred by mothers of taking their children 0.4 times (USD 0.53) more for MV to the costs of MV vaccination in the restrictive MV policy scenario.

### Effect

The effect measures were deaths averted and life years gained (LYG). We calculated deaths averted in four age groups (9–11 months; 12–23 months; 24–35 months; 36–47 months) using the MV coverage rates at 12, 24 and 36 months, the 2013 post-neonatal infant mortality rate of 47.6 deaths/1000 live births and 71.2 deaths/1000 live births in children aged 1–4 years (17.8/1000 live births per year) [21]. We assumed that mortality was 46% (95% CI: 35–55%) lower in children who were vaccinated under the MV-for-all policy compared with MV-unvaccinated children [12]. Incremental LYG were calculated by multiplying deaths averted with the standard life expectancy of 55 years [20]. We discounted future life years by 3% per year as recommended by the WHO [25].

### Sensitivity analyses

On the cost side, we conducted sensitivity analyses by assuming that two (80% wastage) and three (70% wastage) children were vaccinated per vial under the MV-for-all policy. We also assessed at which wastage level the MV-for-all policy became cost-saving. On the effect side, we did sensitivity analyses using a 33% reduction in mortality, which is the effect of MV seen in a large randomized controlled trial from Guinea-Bissau [11]. We also conducted sensitivity analyses using the 35% and 55% boundaries of the confidence interval of the all-cause mortality reduction in the Strategic Advisory Group of Experts on Immunization (SAGE) analysis (46% (35–55%)) [12]. To inform potential decision-makers, we also conducted a sensitivity analysis using only costs incurred by the health system.

Analyses were conducted in STATA version 12.1 (StataCorp, College Station, TX) and Microsoft Excel (Microsoft Corp, Redmond, WA).

## Results

In the 2011 birth cohort under surveillance by BHP, 7.6% of children had died before 9 months of age. Of the children born in 2011, under surveillance by the BHP, 1871 children lived in the restrictive MV clusters and 1978 in the MV-for-all clusters, at the ages of 12–23 months. Seventy-four percent (2862/3849) had their vaccination card inspected between 12 and 23 months of age, 69% (2599/3794) between 24 and 35 months and 62% (2242/3604) between 36 and 48 months. In the MV-for-all clusters, the BHP team vaccinated a median of three children per MV vial used. MV coverage by 3 years of age among children living in the restrictive MV clusters was 84% whereas it was 97% among children living in the MV-for-all clusters (Table 2). Coverage was higher in the MV-for-all clusters at all ages compared with the restrictive MV policy clusters (Table 3). The hospital admission rate in the restrictive MV clusters was 12.5 admissions per 1000 person years. For children in the MV-for-all clusters the hospital admission rate was 8.9 admissions per 1000 person years, 29% (−1–50%) lower than in the restrictive-MV policy clusters (*p* = 0.06). The mean duration of hospital admissions was 7.6 days (SD 8.0).

**Table 2.** Measles vaccine-related costs under the restrictive MV policy and the MV-for-all policy.

	Restrictive MV policy	MV-for-all policy
Vaccine wastage (Vaccine wastage factor [VWF])	40% (VWF = 1.67)	90% (VWF = 10)
MV coverage	84%	97%
MV vial costs (USD)	22,676	157,113
Injection supply costs (USD)	2820	5464
**Total vaccine costs (USD)**	25,497	162,578
Extra household costs of seeking MV (USD)	28,924	
**Total costs of the policy (USD)**	**54,420**	**162,578**
Costs per child vaccinated (USD)	1.19	3.08
Cost-savings due to hospital admissions averted (USD)		84,475

**Table 3.** Years of life gained by age of measles vaccination.

Age group	Children alive in the 2011 birth cohort	Mortality rate (per 1000 live born)	Coverage (restrictive MV clusters)	Coverage (MV-for-all clusters)	Deaths averted*	Life years gained	Discounted life years gained
9–12 months	54,573	0.0130	67%	75%	26	1480	697
12–23 months	53,891	0.0178	67%	75%	35	1871	937
24–35 months	52,967	0.0178	83%	93%	43	2255	1142
36–48 months	52,067	0.0178	84%	97%	56	2827	1447
**Total**			**84%**	**97%**	**160**	**8361**	**4223**

Note: *Calculation for deaths averted: number of children*mortality rate*0.46*coverage difference.

### Costs incurred by the health system

Under the current restrictive MV policy, the total costs of MV amounted to USD 25,497 and the attained coverage was 84% at 36 months of age. Under the MV-for-all policy, costs of MV amounted to USD 162,578 to obtain a coverage of 97% at 36 months of age (Table 2). We estimated that 54,573 children were alive at 9 months of age in the 2011 national birth cohort. Among these children, 626 hospital admissions could be averted under the MV-for-all policy due to higher MV coverage, resulting in a saving of USD 71,165 for the health system.

### Costs incurred by households

When extra trips to the health centre to obtain MV incurred by the household was added to the current restrictive MV policy, the costs of the policy increased to USD 54,420 (Table 3). Adding the value of mother’s time spent accompanying her child to hospital, increased the total cost-saving to USD 84,475 due to averted hospital admissions (Table 2).

### Mortality effects

Assuming a 46% mortality reduction due to higher MV coverage, 160 deaths could be averted under the MV-for-all policy compared with the restrictive MV policy (Table 3). The incremental LYG under the MV-for-all policy compared with the restrictive MV policy amounted to 8361 LYG, and 4223 discounted LYG.

### Cost-effectiveness

Subtracting the savings of hospital admissions averted, the costs of the MV-for-all policy were USD 78,102 (Table 4). The policy was highly cost-effective with an incremental cost-effectiveness ratio of USD 5.61/discounted LYG and USD 148/death averted (Table 4).

**Table 4.** Cost-effectiveness results.

	Costs (USD)	Incremental effects	Incremental cost-effectiveness
**Restrictive MV policy**	54,420	0	0
**MV-for-all policy**	78,102	4223 discounted LYG	5.61 USD/LYG
**MV-for-all policy**	78,102	160 deaths averted	148 USD/death averted

Note: LYG – life years gained.

### Sensitivity analyses

Assuming 80% and 70% wastage, that is, two or three children vaccinated per 10-dose vial, the MV-for-all policy was cost-saving compared with the restrictive MV policy (Table 5). The MV-for-all policy became cost-saving at a wastage level of 88%.

**Table 5.** Sensitivity analyses.

	Costs (USD)	Incremental effects	Incremental cost-effectiveness
**80% MV wastage**			
Restrictive MV policy	54,420	0	Reference
MV-for-all policy	−1779	4223 discounted LYG	Cost-saving
MV-for-all policy	−1779	160 deaths averted	Cost-saving
**70% MV wastage**			
Restrictive MV policy	54,420	0	Reference
MV-for-all policy	−28,406	4223 discounted LYG	Cost-saving
MV-for-all policy	−28,406	160 deaths averted	Cost-saving
**33% reduction in mortality due to MV as obtained from trial [11]**			
Restrictive MV policy	54,420	0	Reference
MV-for-all policy	78,102	3029 discounted LYG	7.8 USD/LYG
MV-for-all policy	78,102	115 deaths averted	206 USD/death averted
**Lower confidence interval boundary from WHO review (35%) [12]**			
Restrictive MV policy	54,420	0	Reference
MV-for-all policy	78,102	3212 discounted LYG	7.4 USD/LYG
MV-for-all policy	78,102	122 deaths averted	194 USD/death averted
**Upper confidence interval boundary from WHO review (55%) [12]**			
Restrictive MV policy	43,506	0	Reference
MV-for-all policy	78,102	5050 discounted LYG	4.7 USD/LYG
MV-for-all policy	78,102	192 deaths averted	124 USD/death averted
**Using only costs incurred by the health system**			
Restrictive MV policy	25,497	0	Reference
MV-for-all policy	91,413	4223 discounted LYG	15.6 USD/LYG
MV-for-all policy	91,413	160 deaths averted	412 USD/death averted

Note: LYG – life years gained.

Assuming a 33% mortality reduction of MV, 115 deaths were averted, the incremental costs per death averted were USD 206 and the costs per discounted LYG were USD 7.8. Assuming 90% wastage and the lower boundary of the confidence interval (35% reduction in mortality), cost-effectiveness was USD 7.4/LYG and USD 194/death averted. Using the upper boundary (55%), cost-effectiveness was USD 4.7/LYG and USD 124/death averted.

Including only costs incurred by the health system, the incremental cost-effectiveness of an MV-for-all policy was 15.6 USD/LYG and 412 USD/death averted. At 83% wastage, MV-for-all was cost-saving when only taking the costs incurred by the health system into account.

## Discussion

MV coverage was significantly lower in the restrictive MV clusters (84%) compared with MV-for-all clusters (97%). The hospital admission rate among children aged 9–48 months in the MV-for-all clusters was 29% lower than for children in the restrictive MV clusters, in line with previous findings [26]. The incremental cost-effectiveness ratio of providing MV to all children compared with the status quo was low (USD 5.61/LYG). For our base-case analysis, we used a worst-case scenario assumption of 90% vaccine wastage (one child vaccinated per vial) under the MV-for-all policy. MV-for-all was cost-saving at 88% wastage and at 83% if only costs incurred by the health system were taken into account. Among children included in the MVEPI study, a median of three children were vaccinated per MV vial in the MV-for-all clusters, giving a wastage rate of 70%.

### Strengths and limitations

The use of primary data is a major strength of this study. We estimated MV coverage under two measles vaccination scenarios using data from a large cluster-randomized trial. Furthermore, all costs were derived from Guinea-Bissau, making the estimates relevant to the setting. Our hospital admission costs were derived from a detailed micro-costing study recently conducted in Guinea-Bissau, from a government perspective [18].

Our study adds important new knowledge since it assesses the cost-effectiveness of a measles vaccination policy taking non-specific effects into account. By assessing the effect of MV on all-cause mortality and not merely on measles infection, we believe that our study provides a measure of the true effect of MV, which is also relevant to settings where measles infection is controlled. It should be noted that there were no reported cases of measles during the study period (2011–2015). We used the meta-estimate derived from a recent WHO review of non-specific effects as the effect estimate [12]. This review comprises all studies of the non-specific effects of MV on child health until 2014, and includes studies conducted in a range of different settings and populations. We therefore believe that the estimate more or less reflects the true effect of MV on child health.

Some limitations must be considered. We achieved a 97% coverage in the MV-for-all clusters using the BHP infrastructure, thus a similar coverage could only be achieved in Guinea-Bissau if outreach is provided to the same extent as the BHP provide (two household visits per year). Nonetheless, the restrictive clusters received the same amount of outreach, the only differences being the vial opening and the age restriction on MV. Furthermore, a wastage rate of 90% in the MV-for-all clusters is a worst-case scenario – among the children included in the MVEPI study, a median of three children were vaccinated per vial in the MV-for-all clusters. Thus, a wastage rate of 70% under the MV-for-all scenario is probably more realistic. Although we included the value of a household’s time when accompanying a child for hospital admission and extra costs of taking a child for MV under the restrictive MV policy, direct household costs were not included. For instance, we did not have information on informal payments made directly to the nurses for vaccinating a child at the health centre and medical expenses paid out-of-pocket by the household. Adding out-of-pocket medical expenses would increase the cost of hospital admissions, making the MV-for-all policy even more cost-effective. We did not make assumptions about hospital admissions or deaths averted beyond four years of age, although there are studies indicating that the non-specific effects of MV may last longer [27]. Mortality effects of non-specific herd immunity due to increased coverage of MV and lower infectious pressure were also not taken into account.

Although we have assessed the cost-effectiveness of MV using a wide variety of costs and effectiveness measures, preventing illness and death is associated with more far-reaching benefits. Healthy adults have higher labour productivity, which may boost economic development [28]. Vaccines may also increase school attendance and educational attainment, and contribute to declines in the fertility rate following improved child survival [28–30]. Thus, MV is probably much more cost-effective due to the wider effects of improved child health and survival than is captured in the present study.

### Consistency with other studies

Several studies have assessed the cost-effectiveness of MV. In a study from the WHO identifying the most cost-effective child health interventions in sub-Saharan Africa, increasing MV coverage was highly cost-effective. Thus, international USD 29/DALY were averted in a scenario with 80% coverage and international USD 58/DALY were averted in a scenario with 95% coverage [7]. However, many studies assessing the cost-effectiveness of routine MV were conducted in connection with the introduction of MV in the mid-1980s in low-income countries [31]. Due to declines in measles mortality, the cost-effectiveness estimates in the older studies no longer seem relevant for the present setting. Furthermore, we assessed the non-specific effects of MV on all-cause mortality. According to the current evidence of non-specific effects of MV, the main effect of MV on child survival is due to the non-specific effects [32]. Thus, cost-effectiveness studies that only include the effect of measles infection may underestimate the true effect of MV, making the results of our study difficult to compare with the results obtained in traditional cost-effectiveness studies.

### Implications

The WHO recommends 95% MV coverage to control measles infections [2]. Even though only the targeted effects of MV are taken into account, scaling up routine vaccinations in accordance with the goals of the Global Immunization Vision and Strategy (GIVS) [33] was estimated to be very cost-effective: requiring additional USD 0.5 per capita to realize, routine vaccination scale-up is considered one of the cheapest child health interventions in Africa [34].

Scale-up of vaccinations, however, stands in contrast to the strict wastage targets imposed by the EPI programme in Guinea-Bissau [14,35] to fulfil the wastage targets made by GAVI and UNICEF [35]. In general, there are two strategies to reduce open vial vaccine wastage: reduce vial size or increase vaccination session size [36]. In Guinea-Bissau, the latter has been the strategy and the restrictive MV policy is in part a result thereof. Introducing one-dose MV vials as a strategy has to our knowledge not been considered in Guinea-Bissau. In a simulation study of measles vial size from Niger, decreasing vial size from 10-dose to 1-dose vials increased the total volume of vials, cold chain and safe injection equipment, thereby increasing the costs of vaccine administration and waste disposal, which far outweighed the costs saved from the decrease in wasted doses [37]. The cost of single-dose vials was 0.77 USD in 2003 [22]. Hence, the price of vaccinating children using 10-dose vials is lower if only 3 doses are used from each 10-dose MV vial. In the present study, vaccinating more than one child per vial (or 88% wastage) was cost-saving. Thus, the cheapest and simplest way to increase MV coverage is to abolish the restrictive MV policy and open a MV vial for every child irrespective of age and session size.

## Conclusions

One of the major challenges in controlling measles is to increase MV coverage. With this study, we propose that one simple and cheap way of increasing coverage is to remove restrictions imposed by wastage targets and age limits. We have shown that in a setting with biannual home visits, removing wastage targets and the age limit could achieve a coverage of up to 97%, which is in line with the recommendations of the WHO to control measles [38]. Taking the low cost of MV and the marked beneficial effects associated with MV into consideration, we recommend that a 10-dose MV be reclassified as a ‘1+ dose vial’, which is opened for a single child, irrespective of age, but which can be extended to vaccinate up to 10 children.

## Paper context

Measles vaccination is a very cost-effective child health intervention, aimed at reducing measles infections and deaths. Taking non-specific effects of the measles vaccine into account, we estimated the incremental cost-effectiveness of providing measles vaccination for all children in Guinea-Bissau. Abandoning strict measles vaccine wastage policies was very cost-effective in Guinea-Bissau and potentially even cost-saving. Policy makers should take non-specific effects of vaccines into account when calculating cost-effectiveness.
